# Remission of Psychosis in Treatment-Resistant Schizophrenia following Bone Marrow Transplantation: A Case Report

**DOI:** 10.3389/fpsyt.2017.00174

**Published:** 2017-09-21

**Authors:** Tsuyoshi Miyaoka, Rei Wake, Sadayuki Hashioka, Maiko Hayashida, Arata Oh-Nishi, Ilhamuddin Abdul Azis, Muneto Izuhara, Keiko Tsuchie, Tomoko Araki, Ryosuke Arauchi, Rostia Arianna Abdullah, Jun Horiguchi

**Affiliations:** ^1^Department of Psychiatry, School of Medicine, Shimane University, Izumo, Japan

**Keywords:** schizophrenia, bone marrow transplantation, acute myeloid leukemia, curative treatment, immune alterations, cellular therapy, maternal immune activation

## Abstract

The authors present the case of a 24-year-old male with treatment-resistant schizophrenia, with predominant severe delusion and hallucination, who received bone marrow transplantation (BMT) for acute myeloid leukemia. After BMT, he showed a remarkable reduction in psychotic symptoms without administration of neuroleptics. He also showed drastic improvement in social functioning. Follow-up evaluations 2 and 4 years after BMT showed persistent significant improvement of the psychotic state and social functioning. Recent findings show that the major underlying pathogenic mechanism of schizophrenia is immune dysregulation. Thus, conceptually, BMT, a cellular therapy, that facilitates the counteractive processes of balancing inflammation by immune regulation, could produce beneficial clinical effects in patients with treatment-resistant schizophrenia. Further studies are required to define the true benefits of BMT for the possible curative treatment of schizophrenia.

## Background

Increasing evidence suggests a correlation between schizophrenia and immune system disturbances. Genome-wide association studies for linkages with schizophrenia have revealed that the odds ratio is frequently high in immune-related regions among many schizophrenia-related genome loci of patients (1–3). Although schizophrenia is regarded as a syndrome with different biological backgrounds, involvement of immune system disturbances could be one of the common mechanisms.

The association between maternal infection and neurodevelopmental disorders is long standing but not without controversy. After the 1964 rubella pandemic, the incidence of schizophrenia rose from less than 1% in the unexposed population to about 20% in the exposed population (4). Subsequent studies charting historic outbreaks of flu, measles, mumps, chickenpox, and polio have revealed an association with schizophrenia (5). However, not all ecological studies have replicated these associations (6). The differing conclusions may stem from differences in estimating the exposed population (6). Nevertheless, several prospective studies following birth cohorts (7, 8) have consistently revealed an association between maternal viral infection and psychiatric disorders in offspring and added other classes of pathogens to the list: namely, bacterial infections—including pneumonia, sinusitis, and tonsillitis—and the parasite *Toxoplasma gondii* (7, 9).

How can such a diverse group of pathogens confer similar risks of psychotic disorder? Common to the implicated pathogens is the maternal immune response. In support of this possibility, enduring fevers above a certain threshold pose the greatest risk (10). It follows that immune system activation above that threshold due to any environmental insult or genetic predisposition would also increase the risk. Indeed, maternal autoimmune disorders, allergies, asthma, acute stress, and exposure to environmental pollutants—all of which lead to elevated immune responses—have been linked to an enhanced risk of schizophrenia (7, 8). These findings may help to contextualize two recent prospective studies that failed to find a significant association between prenatal infection and schizophrenia after adjusting for parental infection in general, parental psychiatric disorder, and socioeconomic status (11, 12).

An accumulative evidence points to the significant role of neuroinflammation and the immune system in the pathophysiology of schizophrenia (13). There are also numerous reports that support the hypothesis that immune activation is a risk for onset of schizophrenia at adulthood (14, 15). Moreover, evidence from genomic (16), blood (17), postmortem (18), and *in vivo* imaging (19) investigations suggests that immune activation is concerned in the pathophysiology of schizophrenia.

In almost all cases, autoimmune diseases are their favorable reaction to immunoablation and saved by bone marrow transplantation (BMT) (20). Investigation in radiation chimeras established that the immunological and hematological systems possess a mutual stem cell (20).

Knowledge of the clinical observation of schizophrenia after BMT would significantly improve our comprehension of the importance of immune system in schizophrenia. Sommer and van Bekkum requested hematologists and psychiatrists to notify them their case reports, and they submitted this request to the relevant expert journals (20).

In this case report, we show that BMT was effective in treatment of treatment-resistant schizophrenia with predominantly delusion and hallucination symptoms. To the best of our knowledge, this is the primary case observation of successful therapy of treatment-resistant schizophrenia with BMT.

## Case Presentation

The patient was a 24-year-old male. His birth was ordinary, and he grew as normal. After he had graduated from university, he labored in a corporation. His level of social skill was standard. There was not any description of alcohol or drugs use or seizures of epilepsy. In his family, there is nobody with psychiatric and developmental disorders. When the patient was 23 years old, he suffered from insomnia, irritability, and anxiety. In addition, he developed into agitated and spoke incoherently, and persecutory delusions and paranoid ideation arose. Problems of consciousness and convulsions were not detected. He visited the Department of Psychiatry of Shimane University Hospital. Assessment of his psychiatric status confirmed auditory hallucination, suspiciousness, active social avoidance, persecutory delusion, and decline in the social function. His diagnosis was “paranoid schizophrenia” according to DSM-IV-TR (21). Physical and neurological examinations revealed no marked abnormalities. There is no abnormal finding in routine laboratory investigations of serum and urine. In electric encephalography, computed tomography, and magnetic resonance imaging of the brain, there is no abnormality. Administration of quetiapine (QTP) (300 mg/day) was started. One week later, his auditory hallucinations, suspiciousness, active social avoidance, persecutory delusion, and deterioration in the level of social functioning continued. Because he refused to take neuroleptics, the patient’s family managed antipsychotics for him and confirmed that he took antipsychotics. However, significant worsening of his psychiatric symptoms followed. Administration of risperidone (RIS) (12 mg/day) and olanzapine (20 mg/day) was added to QTP. However, his psychotic symptoms were not improved at all. His social functioning also deteriorated. Treatment-resistant schizophrenia was classified as little or no response to treatment from at least two adequately dosed antipsychotic trials for least 4 weeks including at least one second-generation antipsychotic (22). As result, he was diagnosed with treatment-resistant schizophrenia (23).

When he was 24 years old, he experienced severe tiredness, continuing elevation of fever, pain of general joint, gingival bleeding, and shortness of breath. As result of further examinations, he was diagnosed with acute myeloid leukemia at the Department of Hematology of Shimane University Hospital. His willingness to receive BMT was confirmed; however, the problem of whether he could stand the considerable psychological pressure of BMT, particularly throughout the isolation phase, was not obvious. To elucidate this, a test isolation was performed for 7 days. While his severe auditory hallucinations, suspiciousness, and persecutory delusion continued, severe psychomotor excitement was not recognized. Moreover, the hospital staff could communicate with him with no difficulty. So that hematologists and we judged that, he would be able to tolerate the stress during the isolation period. All neuroleptics were stopped during the test isolation in the germ-free unit.

One week later, BMT was performed. He was treated in isolation room at germ-free unit for 34 days. We met him three times a week throughout the isolation phase to assessment his psychiatric status and necessity of administering additional therapy. No neuroleptics were administered because of his refusal to take them; however, his psychotic status maintained with stable condition. Moreover, the BMT isolation was accomplished with no trouble. After he underwent BMT, administration of methotrexate and cyclosporin A was begun to avoid graft versus host disease (GVHD). Three weeks after BMT, early symptoms of GVHD were recognized, and hematologists administered tacrolimus in place of cyclosporin A.

**Figure 1:**
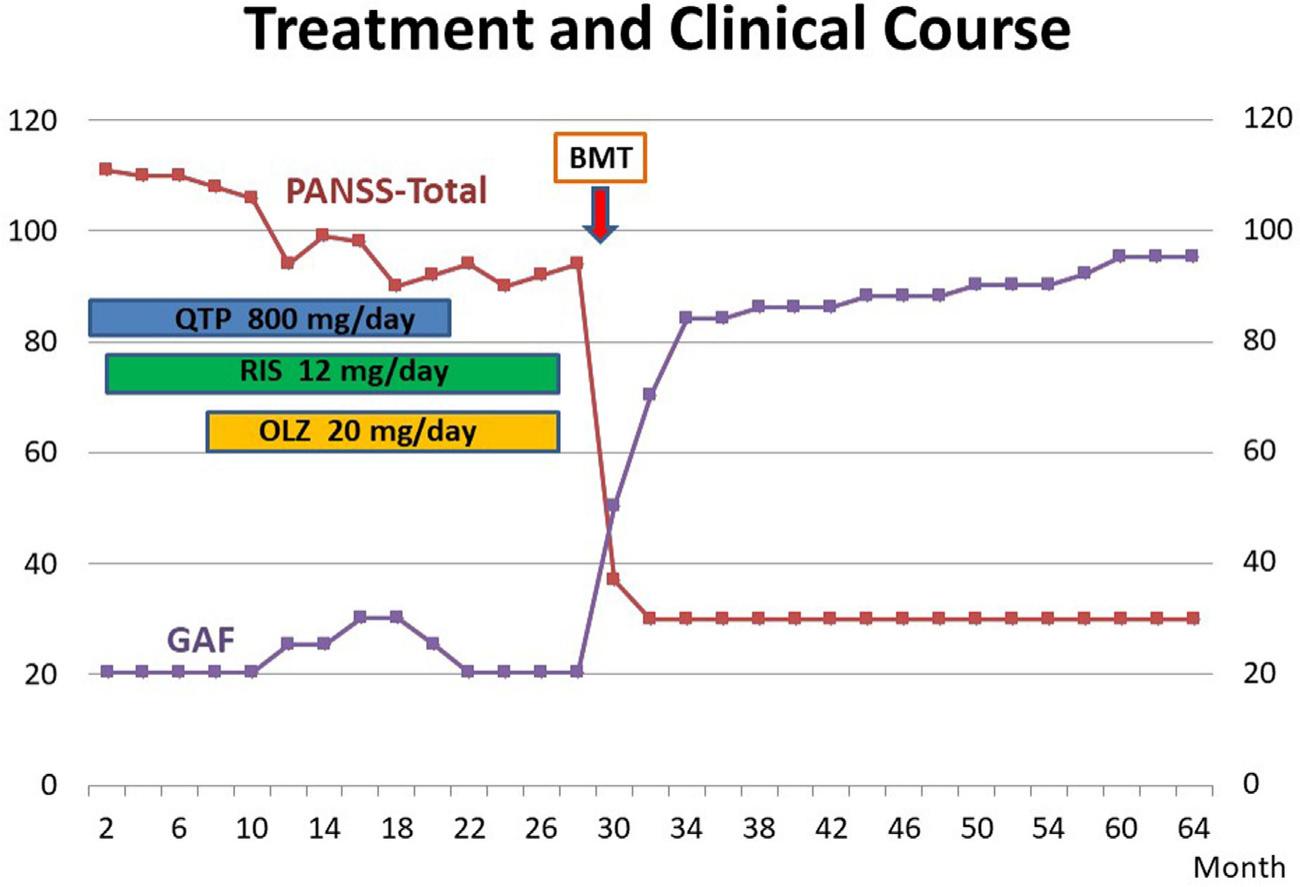
Treatment and clinical course of the case. Their psychiatric symptoms were evaluated by PANSS (24). Functioning was assessed using the GAF of the DSM-IV-TR (21). BMT, bone marrow transplantation; QTP, quetiapine; RIS, risperidone; OLZ, olanzapine; PANSS, Positive and Negative Symptom Scale; GAF, Global Assessment of Functioning Scale.

Thirty days later, his psychotic symptom had almost disappeared. He was sustained without any neuroleptic treatment and need for any other administration. His psychiatric status was assessed by the Positive and Negative Symptom Scale (24). Social functioning was assessed using the Global Assessment of Functioning Scale of the DSM-IV-TR (21). The treatment and clinical course are shown in Figure 1. In 2017, 8 years after BMT, the improvements of somatic and psychiatric symptoms are continued, and the patient is very well and there are no residual psychiatric symptoms. Moreover, his social functioning was drastically recovered, and he continues to work at a famous company.

## Discussion

Bone marrow transplantation might be effective in treatment of this patient’s acute and treatment-resistant schizophrenia characterized predominantly by delusion and hallucination. During the remission of psychosis, this patient did not experience any infection by BMT. To the best of our knowledge, this is the primary case observation of successful therapy of schizophrenia with BMT. In limitation, we could not exclude the possibilities of spontaneous improvement without any treatment, paradoxical improvement following cessation of neuroleptics, and the curative effect of multiple immune modulating drugs.

In consideration of single case report, we apparently cannot confirm an immune pathogenesis of schizophrenia. However, several reports support the theory that immunological system is one of key factor of pathogenesis of schizophrenia (25, 26), and we suggest that physicians and patients involved in BMT consider the possibility that schizophrenia may be treated successfully by BMT.

In an animal study using maternal immune activation (MIA) offspring, Hsiao et al. identified distinction in immune activation in a mouse model of autism and schizophrenia (27). MIA in pregnant rodents can be produced by immunological activation by polyriboinosinic-polyribocytidilic acid (Poly I:C), which causes the offspring to have enduring immune system abnormalities and behavioral abnormalities (9, 27–30). Moreover, it was reported that Poly I:C-induced MIA leads to permanently hyperresponsive CD4+ T cells and a hypersensitive immune system in offspring, and further, that behavioral abnormalities of the rodents could in part be recovered by BMT (27).

Our findings may be contributed to several number of animal model studies reporting the efficacy of BMT on improving symptoms of neurological disorders (31–33). Derecki et al. identified microglia normalized by BMT contributed to recover behavioral abnormalities in a mouse model of Rett syndrome. The findings suggest that BMT normalizes microglia impairments in the brain. Microglia impairment seems to be one important neurological pathology in schizophrenia patient brain (34, 35). However, if only microglia-mediated mechanism is being considered, it is difficult to explain the mechanism *via* which BMT would lead to sudden reversal of symptoms in this case.

In a human clinical case report, Sommer et al. reported the clinical course of a patient who showed severe psychosis after BMT from schizophrenic patients (36). This report also supports the possibility that BMT might be an effective treatment for schizophrenia (37).

Additional research with added subjects is obviously necessary because the association of both schizophrenia and the contribution of BMT in CNS are not comprehended at all.

## Concluding Remarks

In this patient, BMT was effective in treatment of acute and treatment-resistant schizophrenia with predominant delusion and hallucination. During the remission of psychosis, this patient did not experience any infection-associated BMT. In consideration of single case report, we apparently cannot confirm an immune pathogenesis of schizophrenia. However, several reports support the theory that immunological system is one of key factor of pathogenesis of schizophrenia (26), and we suggest that physicians and patients involved in BMT consider the possibility that treatment-resistant schizophrenia may be treated curatively by BMT. Though BMT may not be a cure for all cases of schizophrenia, it definitely possesses the potential to manage overall disease severity and improve the quality of life, and this case report is a preliminary demonstration of the safety and efficacy of BMT in treatment-resistant schizophrenia. Additional research with added subjects is obviously necessary because the association of both schizophrenia and the contribution of BMT in CNS are not comprehended at all.

## Ethics Statement

This case study was carried out in accordance with the recommendations of the Ethical Committee of Shimane University Faculty of Medicine with written informed consent from the subject. The subject gave written informed consent in accordance with the Declaration of Helsinki.

## Author Contributions

Substantial contributions to the conception or design of the work: TM, JH, RW, SH, MH, and MI. The acquisition, analysis, or interpretation of data for the work: TM, IA, KT, TA, RA, and RAA. Drafting the work or revising it critically for important intellectual content: TM, RW, SH, AO-N, and JH. Final approval of the version to be published: TM, JH, RW, SH, AO-N, IA, KT, TA, RA, and RAA.

## Conflict of Interest Statement

The authors declare that the research was conducted in the absence of any commercial or financial relationships that could be construed as a potential conflict of interest.
